# Comparative Genome Mapping Between Chinook Salmon (*Oncorhynchus tshawytscha*) and Rainbow Trout (*O. mykiss*) Based on Homologous Microsatellite Loci

**DOI:** 10.1534/g3.113.008003

**Published:** 2013-10-29

**Authors:** Kerry A. Naish, Ruth B. Phillips, Marine S. O. Brieuc, Lyndsay R. Newton, Anna E. Elz, Linda K. Park

**Affiliations:** * School of Aquatic and Fishery Sciences, University of Washington, Seattle, Washington, 98195; † Washington State University-Vancouver, Vancouver, Washington, 98686; ‡ National Oceanic and Atmospheric Administration, National Marine Fisheries Service, Northwest Fisheries Science Center Conservation Biology Division, Seattle, Washington 98112

**Keywords:** Chinook salmon, *Oncorhynchus tshawytscha*, linkage map, microsatellite, comparative mapping

## Abstract

Comparative genome mapping can rapidly facilitate the transfer of DNA sequence information from a well-characterized species to one that is less described. Chromosome arm numbers are conserved between members of the teleost family Salmonidae, order Salmoniformes, permitting rapid alignment of large syntenic blocks of DNA between members of the group. However, extensive Robertsonian rearrangements after an ancestral whole-genome duplication event has resulted in different chromosome numbers across Salmonid taxa. In anticipation of the rapid application of genomic data across members of the Pacific salmon genus *Oncorhynchus*, we mapped the genome of Chinook salmon (*O. tshawytscha*) by using 361 microsatellite loci and compared linkage groups to those already derived for a well-characterized species rainbow trout (*O. mykiss)*. The Chinook salmon female map length was 1526 cM, the male map 733 cM, and the consensus map between the two sexes was 2206 cM. The average female to male recombination ratio was 5.43 (range 1−42.8 across all pairwise marker comparisons). We detected 34 linkage groups that corresponded with all chromosome arms mapped with homologous loci in rainbow trout and inferred that 16 represented metacentric chromosomes and 18 represented acrocentric chromosomes. Up to 13 chromosomes were conserved between the two species, suggesting that their structure precedes the divergence between Chinook salmon and rainbow trout. However, marker order differed in one of these linkage groups. The remaining linkage group structures reflected independent Robertsonian chromosomal arrangements, possibly after divergence. The putative linkage group homologies presented here are expected to facilitate future DNA sequencing efforts in Chinook salmon.

The feasibility of genome-wide studies in nonmodel organisms has improved with the rapid development of genome-sequencing technologies. Evolutionary studies, for example, have been enhanced by comparing sequence data between nonmodel and model species whose genomes have been fully sequenced (Kim *et al.* 2013; Pfeifer *et al.* 2013; Rands *et al.* 2013; Song and Wang 2013). Comparative genomics can be accelerated in cases in which chromosomal affinities between species are known in advance, because it is possible to align syntenic blocks of DNA sequences across chromosomes, estimate genome coverage within nonmodel species, and rapidly locate loci of phenotypic significance.

Within fishes, the family Salmonidae, order Salmoniformes, is descended from a teleost lineage that underwent a whole-genome duplication event, estimated to have occurred 25−100 million years ago (Allendorf and Thorgaard 1984). Although the genomes of these species are returning to a diploid state, signatures of the tetraploid origin are evident in both the karyotype and the genome (Wright *et al.* 1983). Chromosome arm number and genome size is approximately double that of related freshwater fishes (Phillips and Rab 2001), the Esocidae, and duplicated loci have been extensively reported in all species. Observations of multivalent pairings between homologous and homeologous chromosome arms during meiosis and evidence of tetrasomic inheritance have motivated a model of secondary tetrasomy for male salmonids (Wright *et al.* 1983; Allendorf and Danzmann 1997).

Although chromosome arm number (NF) is conserved across the subfamily Salmoninae, chromosome number varies (Phillips and Rab 2001; Phillips *et al.* 2009). Robertsonian centric translocations or fissions are common, and largely explain the distribution of metacentric and acrocentric chromosomes across species. Whole-genome sequencing of two species, Atlantic salmon *Salmo salar* (Davidson *et al.* 2010) and rainbow trout *Oncorhynchus mykiss* (Govoroun *et al.* 2006; M. R. Miller, personal communication), are underway. Given that whole chromosome arms are syntenic between salmonids, aligning their chromosomes through genetic and physical mapping will facilitate the rapid application of sequence information in rainbow trout and Atlantic salmon to other related species. Furthermore, the evolution of chromosome organization and structure subsequent to the whole-genome duplication event can be characterized.

Genome maps for a number of salmon species have been developed (*e.g.*, Lindner *et al.* 2000; Sakamoto *et al.* 2000; Nichols *et al.* 2003; Gilbey *et al.* 2004; Woram *et al.* 2004; Gharbi *et al.* 2006; Guyomard *et al.* 2006; McClelland and Naish 2008; Moen *et al.* 2008; Palti *et al.* 2012). However, extensive comparisons of syntenic relationships between chromosomes and linkage groups have been largely focused between genera within the Salmoninae [*Salmo*, *Oncorhynchus*, and *Salvelinus* (Danzmann *et al.* 2005; Phillips *et al.* 2009; Lien *et al.* 2011)] and between salmonids and other fishes (Rexroad *et al.* 2005; Danzmann *et al.* 2008; Guyomard *et al.* 2012). These latter studies have comprehensively described whole-chromosome arm homologies between rainbow trout and Atlantic salmon, and extensive linkage group affinities between the ancestral ray-finned fishes and the duplicated chromosomes in salmon species. Here, we focus on more recent evolutionary events in chromosome organization by reporting the results of comparative mapping between Chinook salmon *O. tshawytscha* and rainbow trout, two species within the Pacific salmon and trout genus, *Oncorhynchus*.

There are several species within *Oncorhynchus*, a genus that includes anadromous and freshwater life histories. Chromosome arm number within this genus is approximately 100, but the diploid chromosome number varies between 52 and 74 (Phillips and Rab 2001). This variation is explained by differences in the number of acrocentric and metacentric chromosomes among species. A comparison within the genus will provide insight into the processes involved in recent chromosome evolution, as well as extend genomic resources to a less studied species, Chinook salmon.

The aim of our research was to derive a linkage map for Chinook salmon and to compare this map with that of rainbow trout so that we could study chromosome rearrangements within the genus *Oncorhynchus*. We used microsatellite loci, many of which are conserved across multiple species, because they are highly variable and have been extensively mapped in rainbow trout. Indeed, such multiallelic markers are useful in comparative mapping efforts because they are more likely to be polymorphic across species than single nucleotide polymorphisms detected by newer approaches, namely genotyping by sequencing. We explicitly targeted loci mapped in Rexroad *et al.* (2008) and Guyomard *et al.* (2006), because the chromosome arm and centromere position is known in these maps. We could therefore identify the corresponding chromosome arms in Chinook salmon. The derivation of the Chinook salmon map will permit alignment of linkage groups to chromosomes through physical mapping (Phillips *et al.*, 2013, accompanying paper), and advance genome characterization efforts in the species as a whole.

## Methods

### Reference mapping panel

A within-population outbred cross was created by mating a single F1 male with one F1 female from Grovers Creek hatchery population within the Puget Sound in Washington State (location: 47.791016°N, 122.557983°W). Fin clips from the F0 grandparents, the F1 male and female parents, and all F2 offspring from the resulting family were collected at sexual maturity and stored in ethanol. Whole tissues of F1 parents and F2 offspring were maintained at −80°. All individuals were sexed visually by inspecting maturing gonads. DNA was extracted from the fin tissue of F0, F1, and 48 F2 offspring following manufacturer’s recommendations (DNA easy kit; QIAGEN).

### Molecular analyses

All individuals were genotyped by the use of allozyme loci (Aebersold *et al.* 1987) and microsatellite markers developed in salmon species (microsatellite loci are given in Supporting Information, Table S1). Although we used a range of loci identified from the literature and from databases, we specifically screened microsatellite markers that have been previously mapped to known chromosome arms in *O. mykiss* (Guyomard *et al.* 2006; Rexroad *et al.* 2008).

*Microsatellite loci* were amplified using one of three protocols, all of which were interchangeable between loci. The first comprised approximately 30 ng of genomic DNA, 1× buffer (10 mM Tris-Cl, 50 mM KCl, 0.1% Triton X-100), 1.0–2.5 mM MgCl_2_, 200 µM each dNTP, 2 pmol each of a labeled forward and reverse primers, and 0.5 units of *Taq* (GeneChoice) in 10-µL reactions. The second used the primer tailing protocol of Schuelke (2000), comprising the same reaction conditions as noted previously, except for 2.0 mM or 2.5 mM MgCl_2_, 1.5 pmol of labeled M13 forward primer, 1.5 pmol of locus-specific reverse primer, and 0.16 pmol of locus-specific forward primer. The third relied on QIAGEN’s Multiplex PCR mix, where we used 2.0 pmol of labeled M13 forward primer, 2.0 pmol of locus-specific reverse primer, 0. 5 pmol of locus-specific forward primer, 0.9× PCR Master mix and 30−60 ng of DNA in a 5- or 10-µL reaction.

All reactions were carried out using an MJ Thermocycler. Amplifications using labeled primers involved one cycle of denaturation at 95° for 15 min, and 29 cycles of 95° for 30 sec, primer-specific annealing temperature for 90 sec (Table S1), and extension at 72° for 60 sec. Amplification reactions using M13-labeled primers used an initial denaturation at 95° for 15 min, and 29 cycles of 94° for 30 sec, primer-specific annealing temperature (Table S1) for 90 sec, and extension at 72° for 60s, followed by seven cycles of 94° for 30 sec, 53° for 90 sec and extension at 72° for 60 sec, with a final extension at 60° for 30 min. Fragments were visualized with a MegaBACE 1000 capillary electrophoresis system (GE Healthcare), and genotypes were scored using GENE PROFILER software (GE Healthcare).

*Allozyme loci* were identified using starch gel protein electrophoresis, following the protocols outlined in Aebersold *et al.* (1987). The following loci were screened for segregating polymorphisms; MPI*, sSOD-1*, SIDH-2P*, and GTH-2B*.

### Linkage mapping

Sex-specific maps were generated using LINKMFEX V2.3 (Danzmann 2005) to accommodate significant differences in recombination rates between males and females, frequently observed in salmonids. Here, the pairwise recombination fraction between markers, *θ*, was used to estimate map distances because there is almost complete interference during meiosis in salmonids, resulting in one or no crossover events on chromosome arms (Thorgaard *et al.* 1983). A linkage disequilibrium LOD score of 3.0 was used to cluster markers initially, but consensus marker order within linkage groups was determined using MAPORD within LINKMFEX using an LOD of 4.0. Recombination ratios between females and males were compared with RECOMDIF within LINKMFEX. Consensus maps between the sexes were produced in ONEMAP (Margarido *et al.* 2007) using Kosambi mapping distances (*θ* is not given in this package) and an LOD of 4.0. Framework markers comprising loci that were polymorphic in both sexes were used to construct initial orders, and remaining markers were added sequentially. Marker order was verified using the “ripple” function in ONEMAP. Graphical representation of maps was produced using MAPCHART (Voorrips 2002).

### Nomenclature and comparative mapping

Linkage group names (designated “Ck”) were assigned randomly. These groups were subsequently aligned with Chinook chromosome arms in an accompanying publication (Phillips *et al.* 2013). Marker nomenclature followed published conventions (Nichols *et al.* 2003; Danzmann *et al.* 2008). Where possible, specific *O. mykiss* chromosome arm designators were assigned to individual markers on the basis of their positions in Guyomard *et al.* (2006) and Rexroad *et al.* (2008) following comparison with Phillips *et al.* (2006). These markers were used to identify likely chromosome arm placement within Chinook linkage groups.

## Results

### Linkage analyses and sex-specific recombination differences

We examined segregation in 364 polymorphic molecular loci (361 microsatellite loci, 3 allozymes) and one sex locus. A total of 33 microsatellite primers amplified pairs of duplicated loci, and 73 loci were known to be linked to expressed sequences (Rise *et al.* 2004; Rexroad *et al.* 2005; Guyomard *et al.* 2006). Original genotypes for each individual are listed in Table S10. Linkage analysis resulted in assignment of 335 loci to 34 linkage groups (Table S2, Table S3, Table S4, and Table S5), although one linkage group (Ck33) was not mapped in the male parent, and another (Ck31) did not share markers between the sexes.

**Figure 1:**
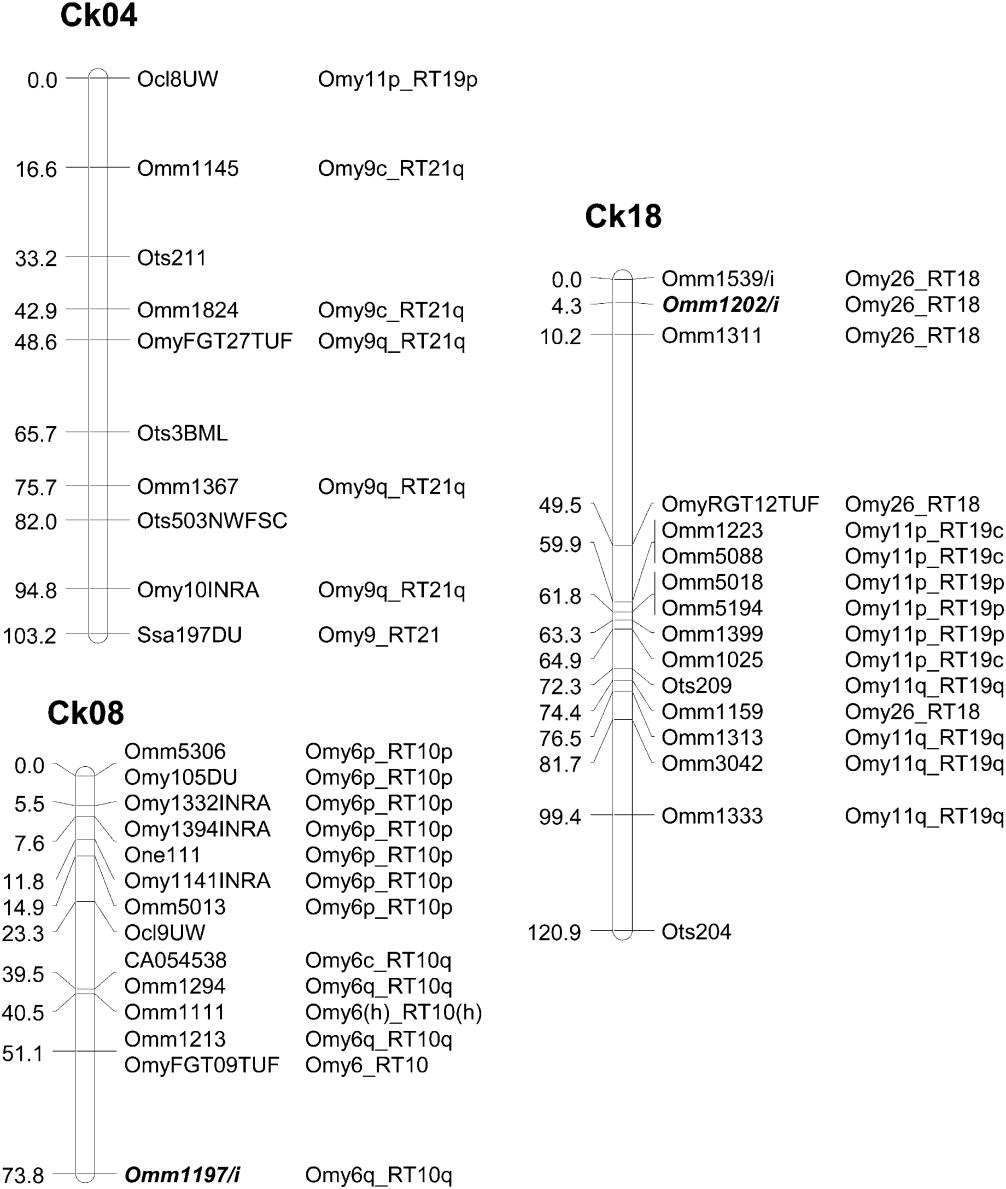
Graphic representation of three linkage groups, Ck04, Ck08, and Ck18 mapped in Chinook salmon. The linkage groups are a consensus of marker order and distances within the female and male maps. Recombination distances are in centiMorgan (Kosambi). Loci are annotated with the chromosome (Omy) or linkage group (RT) assignment in rainbow trout. Rainbow trout chromosome arms (p or q) or centromere positions (c) are identified where possible. Markers that mapped to the homeolog in rainbow trout are designated (h). Duplicated loci (bold) are identified as /i or /ii and annotated with the homeologous Chinook linkage groups.

The female map comprised 261 markers with a map length of 1498 cM Haldanes (1526 cM Kosambi, Table S3 and Table S7), the male map 250 markers with a map length of 733cM (757.4 cM Kosambi, Table S4 and Table S7), whereas the sex-averaged consensus map comprised 317 markers with a map length of 2206.2 cM (Kosambi, Figure 1, Figure S1, Table S2, and Table S6). Some markers could not be accurately placed on the consensus map because of differences in recombination distances between the two sexes, and so the full set of mapped markers was not used in this map.

Like all Salmoninae species reported to date, we noted large differences in recombination rates between the sexes. Assessment of the differences, excluding duplicated markers, revealed average female: male recombination ratios to be 5.43 (range 1−42.8, Table S8) across all pairwise comparisons (n = 344) among markers shared within linked segments (where average recombination ratio = average number of recombinants in female: average number of recombinants in male). This difference was highly significant (G-test value = 2000, 1 d.f.) It was not possible to discern a trend by linkage group structure, because many female linkage groups were incomplete. That said, there were individual linkage groups (Ck03 and Ck19) in which this trend was not supported; female recombination was suppressed relative to male recombination.

### Comparative mapping and linkage group structure

**Figure 2:**
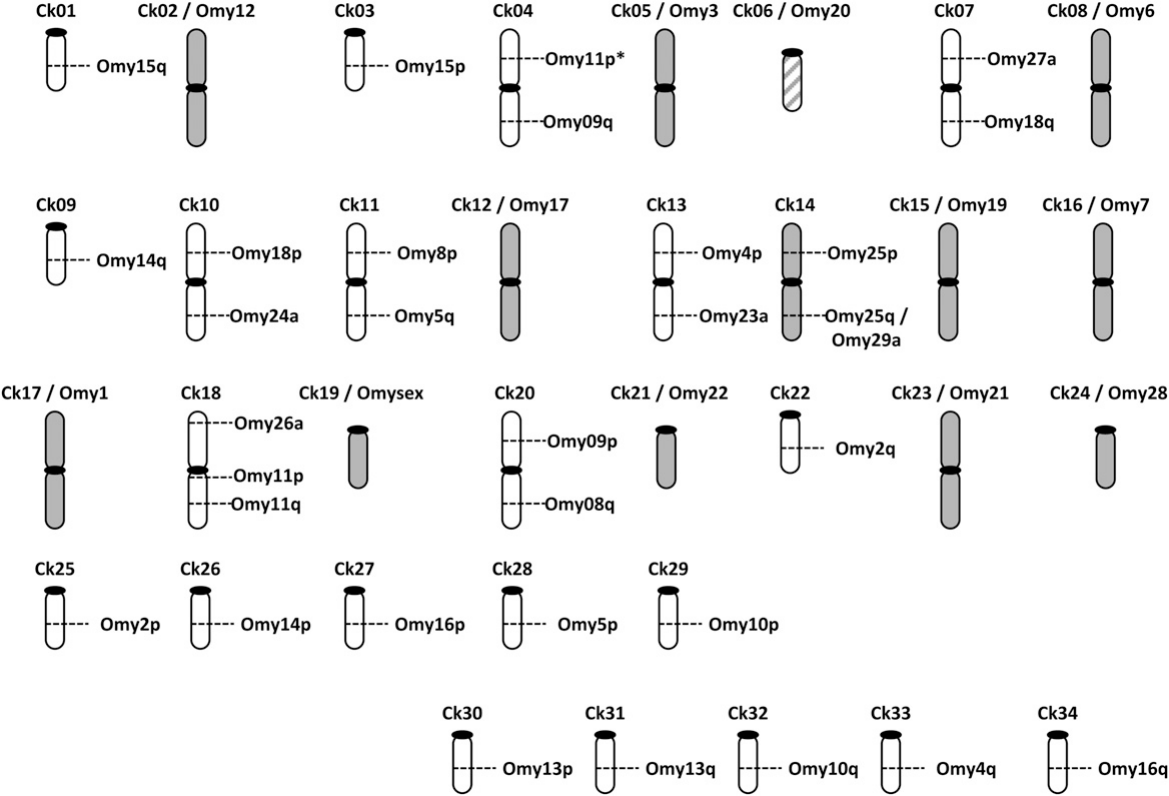
Ideogram of the relationship between Chinook salmon linkage groups (Ck) and Rainbow trout chromosome arms (Omy), based on homologous loci mapped in the two species. Rainbow trout chromosome arms are given as p and q for metacentric chromosomes or a for acrocentric chromosomes. Gray linkage groups are conserved between the two species; marker order is not conserved in Ck06/Omy20. *Designates a postulated metacentric arm in Chinook salmon.

We mapped 283 markers in Chinook salmon that had been previously placed in linkage groups in rainbow trout. Marker annotation with rainbow trout linkage group name (Figure 1, Figure S1, Table S4, Table S5, and Table S6) permitted alignment of linkage groups between the two species and inference of Chinook linkage group structure (Figure 2). Chromosome arm homologies between the two species were assumed where linkage groups shared two or more markers (range 2−10), excluding loci that mapped to the centromere region in rainbow trout. There was one exception—one linkage group (Ck04, discussed below, Discussion paragraph 2) had only one marker from one rainbow trout chromosome arm that mapped to nine markers from a different arm. All rainbow trout chromosome arms were mapped in Chinook salmon, resulting in a haploid arm number of 50. Sixteen linkage groups corresponded to putative bi-armed metacentric chromosomes (including Ck04), and 18 represented putative uni-armed acrocentric chromosomes. These inferred structures agree with the known chromosome arrangements in Chinook salmon (Phillips and Rab 2001).

**Figure 3:**
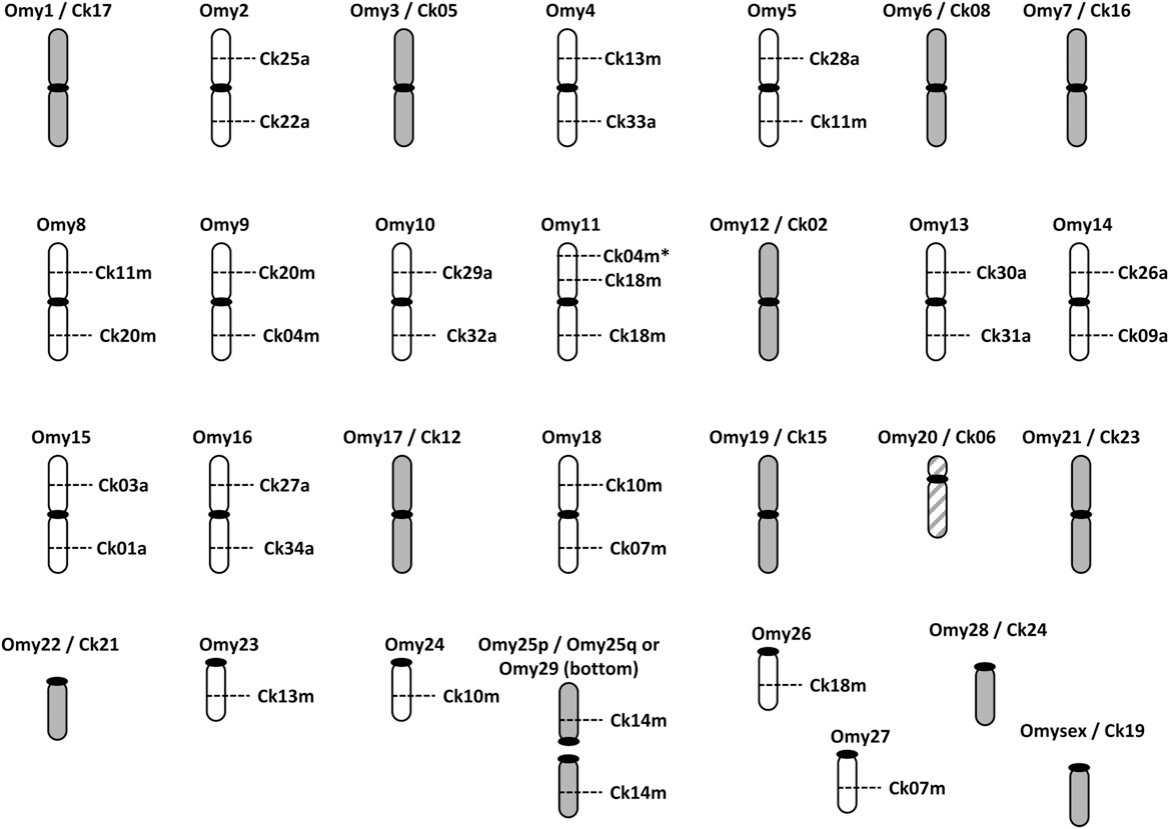
Ideogram of the relationship between Rainbow trout chromosome arms (Omy) and Chinook salmon linkage groups (Ck), based on homologous loci mapped in the two species. Rainbow trout chromosome arms are given as p and q for metacentric chromsomes or a for acrocentric chromosomes. Putative linkage group structure in Chinook salmon is given as m for metacentric and a for acrocentric chromosomes. Gray linkage groups are conserved between the two species; marker order is not conserved in Omy20/Ck06.

Comparative mapping between the Chinook salmon and rainbow trout maps (Figure 2 and Figure 3) suggest that at least 12 chromosomes are conserved between the species. However, chromosome structure in rainbow trout is polymorphic. The linkage map of Rexroad *et al.* (2008) describes 22 metacentric and 9 acrocentric chromosomes, whereas that of Guyomard *et al.* (2006) corresponds to 21 metacentric and 11 acrocentric chromosomes. Omy25 represents one metacentric linkage group in the former, but is described by two acrocentric chromosomes in the latter (Omy25/29). The polymorphic chromosome in rainbow trout, Omy25, is metacentric in Chinook. The arm arrangement is consistent between Chinook salmon and the map of Rexroad *et al.* (2005), providing a 13th chromosome that is conserved in this specific comparison.

The remaining chromosomes reflect Robertsonian rearrangements that occurred independently in the two species since divergence from a common ancestor. Six metacentric chromosomes in rainbow trout (Omy 2, 10, 13, 14, 15, and 16) correspond to 12 acrocentric chromosomes in Chinook salmon (Figure 2 and Figure 3). Three metacentric chromosome pairs in Chinook salmon appear to be the result of centric fusions between arms that are found in two separate metacentric chromosome pairs in rainbow trout (Ck04, Ck11, Ck20). Another three are a fusion between one acrocentric chromosome pair in rainbow trout with another arm that is part of a metacentric chromosome pair (Ck07, Ck10, Ck13).

There are two arrangements that may explain the lower arm number in Chinook salmon (NF = 100) compared with rainbow trout (NF = 104). First, Omy20 is metacentric in rainbow trout, but the p arm likely comprises almost entirely ribosomal RNA genes (Phillips *et al.* 2009). Significantly, we noted a difference in linkage group marker order between the female map for Ck06 and Omy20, explained by an inverted “block” of markers mapping to the q arm in rainbow trout (map distance in female Chinook salmon = 32 cM). This result is consistent with a centromeric inversion in the lineage leading to rainbow trout, resulting in a metacentric chromosome in this species. Therefore, we assumed that this linkage group is acrocentric in Chinook salmon. Second, the smallest metacentric chromosome in rainbow trout (Omy22) corresponds to a chromosome that is likely acrocentric in Chinook salmon (Ck21), the result of an inversion in rainbow trout (Phillips *et al.* 2009).

We also detected a more complex arm rearrangement between the two species. The metacentric chromosome, Ck18, comprises markers from the p and q arms from Omy11. However, Ck18 also includes markers from the acrocentric chromosome, Omy26 (Figure 1). One marker from the telomeric region of Omy11p mapped to the end of Ck04, and thus we speculate that this latter linkage group represents a metacentric chromosome in Chinook salmon, explained by reciprocal translocation, although additional markers from Omy11p would be required to confirm this assumption (Phillips *et al.*, 2013, accompanying paper).

The *sex* locus mapped to a single armed chromosome in Chinook salmon (Ck01). The linkage group assignment for this locus is unique to the species, a result we reported previously (Phillips *et al.* 2005). The Chinook salmon sex linkage group is equivalent to the q arm of Omy15.

### Duplicated regions

We detected nine pairs of duplicated loci where only one of the two loci was polymorphic and placed on the map. An additional 24 pairs of loci were polymorphic at both loci, and of these, both loci for 16 pairs were mapped (Figure S1). In all tables and figures, we identified duplicated loci by /i or /ii, regardless of whether both loci were added to the map. Using the 16 pairs of duplicated loci, we identified nine putative homeologous chromosome arm pairings in Chinook salmon (Table 1). Although four of these pairings were only supported by one marker, all but one pairing (Ck02 with Ck13) agree with previously published rainbow trout maps (summarized in Phillips *et al.* 2006; Danzmann *et al.* 2008; Guyomard *et al.* 2012).

**Table 1:** Putative homeologous relationships between Chinook salmon linkage groups, number of markers supporting relationships, and comparison with homeologous pairings in rainbow trout

Chinook Salmon Linkage Group	Chinook Salmon Homeolog	No. Markers	Rainbow Trout Linkage Group	Rainbow Trout Homeolog
Ck01	Ck23	2*^a^*	Omy15q	Omy21p
Ck02	Ck13	1	Omy12p	Omy23a
Ck02	Ck31	2*^a^*	Omy12q	Omy13q
Ck05	Ck25	3*^a^*	Omy3p	Omy2p
Ck07	Ck26	1*^a^*	Omy18q	Omy14p
Ck08	Ck18	1*^a^*	Omy6q	Omy26a
Ck09	Ck14	1*^a^*	Omy14q	Omy25q/29a
Ck12	Ck30	2*^a^*	Omy17p	Omy13p
Ck15	Ck32	3*^a^*	Omy19p	Omy10q

Linkage groups are given for Chinook salmon, chromosome arms (denoted p, q, or a, acrocentric) for Rainbow trout.

a These pairings have greater support in rainbow trout and Atlantic salmon.

## Discussion

Here, we provided a linkage map for Chinook salmon based on microsatellite markers that permitted alignment with rainbow trout linkage groups. We mapped 34 linkage groups that putatively represent all chromosome arms in the species and inferred chromosome structure for all of these groups. Significant differences in male and female recombination rates, typical of salmonids, were observed. The sex locus mapped to a linkage group representing an acrocentric chromosome. Comparisons between the Chinook salmon and rainbow trout groups revealed several conserved chromosome structures, but also Robertsonian rearrangements that are common in salmonids.

We note some limitations to the interpretation of the results. First, some of the linkage groups in Chinook salmon are represented by a few markers. It is possible that these linkage groups might group with larger ones with the addition of more marker loci, resulting in a revision of the inferred chromosome structure for Chinook salmon (but see Phillips *et al.*, 2013, accompanying paper). Second, the number of individuals mapped was relatively small. The recombination distances and marker orders can be expected to change with the inclusion of additional offspring and markers. Similarly, map distances differed between the comparative map and the female map. This result can be partly explained by the fact that the addition of markers from the male increased map distances and partly because marker orders were difficult to resolve when loci were not polymorphic in both sexes. Third, a number of markers mapped to non-homologous linkage groups between the two species. Most of these differences could be accounted for by examining the homeologous linkage groups in rainbow trout; simply, genotyping efforts in both species might have amplified alternative forms of a duplicated locus. A very small number of differences could not be explained, and might be attributed to error or to the amplification of non-homologous loci. Finally, we concluded that linkage group Ck04 represents a metacentric chromosome, based on a single marker from Omy11p that mapped to this group. Several markers from Omy11p already map to another linkage group, Ck18, along with markers from Omy11q and Omy26. Although it is possible that another linkage group represents a metacentric chromosome, we note that all arms mapped in rainbow trout are accounted for in the Chinook salmon map.

Comparative mapping identified 12−13 chromosomes that are conserved between the two species, depending on the polymorphism observed in one linkage group (Omy25/29) in rainbow trout. It is likely, therefore, that the Robertsonian rearrangements explaining the structures of these chromosomes are ancestral to the divergence of the two species. The most recently phylogenies of the Salmonidae (Crespi and Fulton 2004; Crête-Lafrenière *et al.* 2012) place the common ancestor of the two species at a basal position to all extant species within the genus *Oncorhynchus*. Therefore, it is possible that these chromosome structures are conserved across the genus. Genome maps have also been constructed for coho salmon *O. kisutch* (McClelland and Naish 2008), sockeye salmon *O. nerka* (Everett *et al.* 2012), pink salmon *O. gorbuscha* (Lindner *et al.* 2000), and cutthroat trout *O. clarkii* and rainbow trout hybrids (Ostberg *et al.* 2013). The only linkage map with sufficient marker homology with the two species compared here is that of Ostberg *et al.* (2013), and the same conserved chromosomes are observed in this map, although cutthroat and rainbow trout are sister species (Crespi and Fulton 2004). One chromosome, Omy20/Ck06 is conserved between the Chinook and rainbow trout, but marker order is not. Ostberg *et al.* (2013) suggested that this chromosome underwent a pericentric inversion and is acrocentric in cutthroat trout. We speculate, therefore, that the order difference is due to a centrometric inversion following divergence between rainbow trout and Chinook salmon, and may be exclusive to rainbow trout. The remaining linkage groups are not conserved between Chinook salmon and Rainbow trout, reflecting different rearrangements since divergence from a common ancestor.

Syntenic relationships between the rainbow trout and Atlantic salmon karyotypes, described in Phillips *et al.* (2009), permitted an additional comparison with the Chinook salmon map (Table S9). Atlantic salmon chromosome arm number (NF = 72−74) is reduced compared with those of *Oncorhynchus*, explained by large acrocentric chromosomes that have resulted from tandem fusions (Phillips and Rab 2001). Large blocks in certain large acrocentric chromosomes are homologous with whole arms in rainbow trout (Phillips *et al.* 2009) and, by inference, with Chinook salmon. However, three of the conserved chromosome pairs identified earlier are conserved between all three species and are likely ancestral to the divergence of the two genera. These include a metacentric linkage group AS24 (chromosome Ssa7p,q), Omy21p,q, and Ck23; the acrocentric group AS14 (Ssa21), Omy22, and Ck21; and a group that represents an acrocentric chromosome in Atlantic salmon (AS33, Ssa28) and Chinook salmon (Ck06) and a metacentric chromosome in rainbow trout (Omy20). There are two other relationships of interest. The syntenic block comprising the conserved chromosomes Omy25p,q/Ck14p,q is conserved in the Atlantic salmon acrocentric chromosome AS10 (Ssa9), which has also fused with a third chromosome arm. Finally, Omy11p,q, which corresponds to Ck18 (although part of Omy11p maps to Ck04) is also a conserved block within a large acrocentric chromosome in Atlantic salmon, AS25 (Ssa20qa). Therefore, these blocks might also represent an arrangement ancestral to the divergence of the two genera.

Here, we have presented a first-generation genome map of Chinook salmon that permitted alignment of linkage groups in this species with that of rainbow trout. Microsatellite loci are particularly useful in initial attempts at comparative mapping in species with few genomic resources, because they are conserved between related species and are highly polymorphic (multiallelic). Therefore, these loci are readily placed on genome maps. In the case of rainbow trout and Atlantic salmon in particular, these loci have been used extensively in comparative mapping efforts and have been physically mapped to chromosome arms in all three species (Phillips *et al.*, 2013, accompanying paper). Current maps for salmon species that are based on genotyping by sequencing have not been extensively aligned to chromosome arms (*e.g.*, Everett *et al.* 2012; Houston *et al.* 2012; Miller *et al.* 2012) and could not be used for our purposes. As a result of using microsatellite markers, we have detected a number of chromosome arm arrangements that are likely conserved, proposed putative homologies between remaining chromosome arm rearrangements between the two species, and identified a difference in marker order in one linkage group, possibly a result of an inversion in rainbow trout. This comparison is expected to facilitate future alignment of more extensive DNA sequences generated in Chinook salmon using advanced technologies with those produced in rainbow trout and Atlantic salmon. The development of genomic resources is expected to support future evolutionary and ecological studies, and to facilitate quantitative trait mapping for aquaculture development (Naish and Hard 2008).

Note Added in Proof: See also Ruth B. Phillips, Linda K. Park, and Kerry A. Naish, 2013 Assignment of Chinook Salmon (*Oncorhynchus tshawytscha*) Linkage Groups to Specific Chromosomes Reveals a Karyotype with Multiple Rearrangements of the Chromosome Arms of Rainbow Trout (*Oncorhynchus mykiss*) G3: Genes, Genomes, Genetics 3: 2289–2295.

## Supplementary Material

Supporting Information
