# Supplementary material for: Comparative Genome Mapping Between Chinook Salmon (Oncorhynchus tshawytscha) and Rainbow Trout (O. mykiss) Based on Homologous Microsatellite Loci
Source: G3 (Bethesda). 2013 Oct 29;3(12):2281–8. doi: 10.1534/g3.113.008003 (PMC3852389; doi:10.1534/g3.113.008003)
Supplement: Supporting Information [file supp_3_12_2281__index.html]

Comparative Genome Mapping Between Chinook Salmon (Oncorhynchus tshawytscha) and Rainbow Trout (O. mykiss) Based on Homologous Microsatellite Loci — Supporting Information 

# Comparative Genome Mapping Between Chinook Salmon (*Oncorhynchus tshawytscha*) and Rainbow Trout (*O. mykiss*) Based on Homologous Microsatellite Loci

## Supporting Information for Naish *et al.*, 2013

**Files in this Data Supplement:**

- Supporting Information - Figure S1 and Tables S1-S10 (PDF, 1 MB)
- Figure S1 - Graphic representation of linkage groups mapped in Chinook salmon. (PDF, 610 KB)
- Table S1 - Details on microsatellite loci that were polymorphic in the Chinook salmon mapping family (.zip, 19 KB)
- Table S2 - Assignment of microsatellite markers to Chinook salmon linkage groups (.zip, 20 KB)
- Table S3 - Marker order in the Chinook salmon consensus map (.zip, 21 KB)
- Table S4 - Marker order in the Chinook salmon female map (.zip, 18 KB)
- Table S5 - Marker order in the Chinook salmon male map (.zip, 18 KB)
- Table S6 - Mapchart code for the consensus and sex-specific maps in Chinook salmon (.zip, 15 KB)
- Table S7 - Mapchart code for the consensus and sex-specific maps in Chinook salmon (.zip, 18 KB)
- Table S8 - Female to male recombination ratios between shared markers in each Chinook salmon linkage group (.zip, 4 KB)
- Table S9 - Comparison between Chinook salmon, rainbow trout and Atlantic salmon linkage group or chromosome arm arrangements (.zip, 7 KB)
- Table S10 - Genotypes for parents and offspring in the mapped family, provided for each microsatellite locus (.zip, 41 KB)
