# Supplementary material for: Comparative Genome Mapping Between Chinook Salmon (Oncorhynchus tshawytscha) and Rainbow Trout (O. mykiss) Based on Homologous Microsatellite Loci
Source: G3 (Bethesda). 2013 Oct 29;3(12):2281–8. doi: 10.1534/g3.113.008003 (PMC3852389; doi:10.1534/g3.113.008003)
Supplement: Supporting Information [file supp_g3.113.008003_FigureS1.pdf]

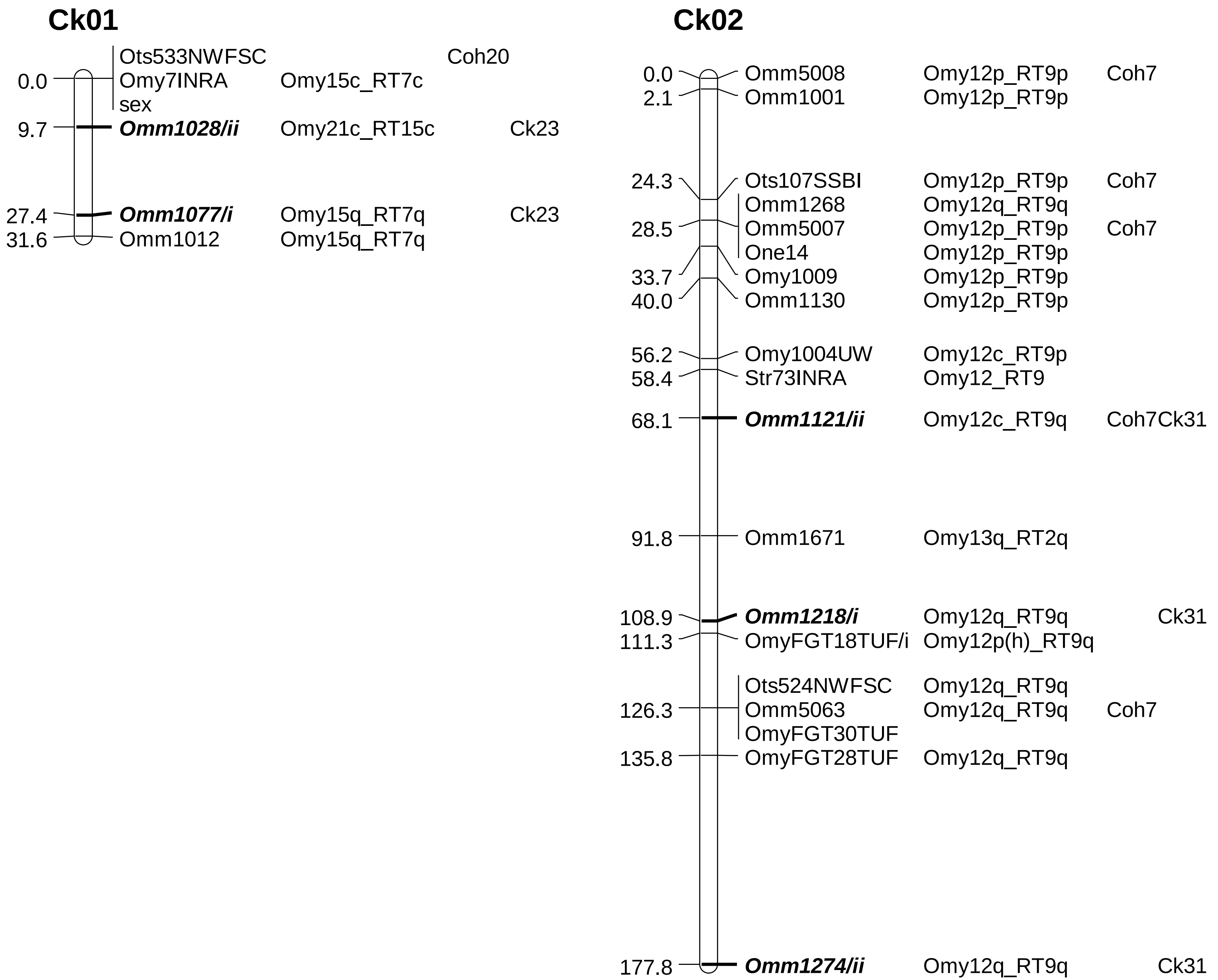
Ck01
0.0 Ots533NWFSC Coh20
Omy7INRA Omy15c_RT7c
sex
9.7 Omm1028/ii Omy21c_RT15c Ck23
27.4 Omm1077/i Omy15q_RT7q Ck23
31.6 Omm1012 Omy15q_RT7q
Ck02
0.0 Omm5008 Omy12p_RT9p Coh7
2.1 Omm1001 Omy12p_RT9p
24.3 Ots107SSBI Omy12p_RT9p Coh7
Omm1268 Omy12q_RT9q
28.5 Omm5007 Omy12p_RT9p Coh7
One14 Omy12p_RT9p
33.7 Omy1009 Omy12p_RT9p
40.0 Omm1130 Omy12p_RT9p
56.2 Omy1004UW Omy12c_RT9p
58.4 Str73INRA Omy12_RT9
68.1 Omm1121/ii Omy12c_RT9q Coh7Ck31
91.8 Omm1671 Omy13q_RT2q
108.9 Omm1218/i Omy12q_RT9q Ck31
111.3 OmyFGT18TUF/i Omy12p(h)_RT9q
Ots524NWFSC Omy12q_RT9q
126.3 Omm5063 Omy12q_RT9q Coh7
OmyFGT30TUF
135.8 OmyFGT28TUF Omy12q_RT9q
177.8 Omm1274/ii Omy12q_RT9q Ck31

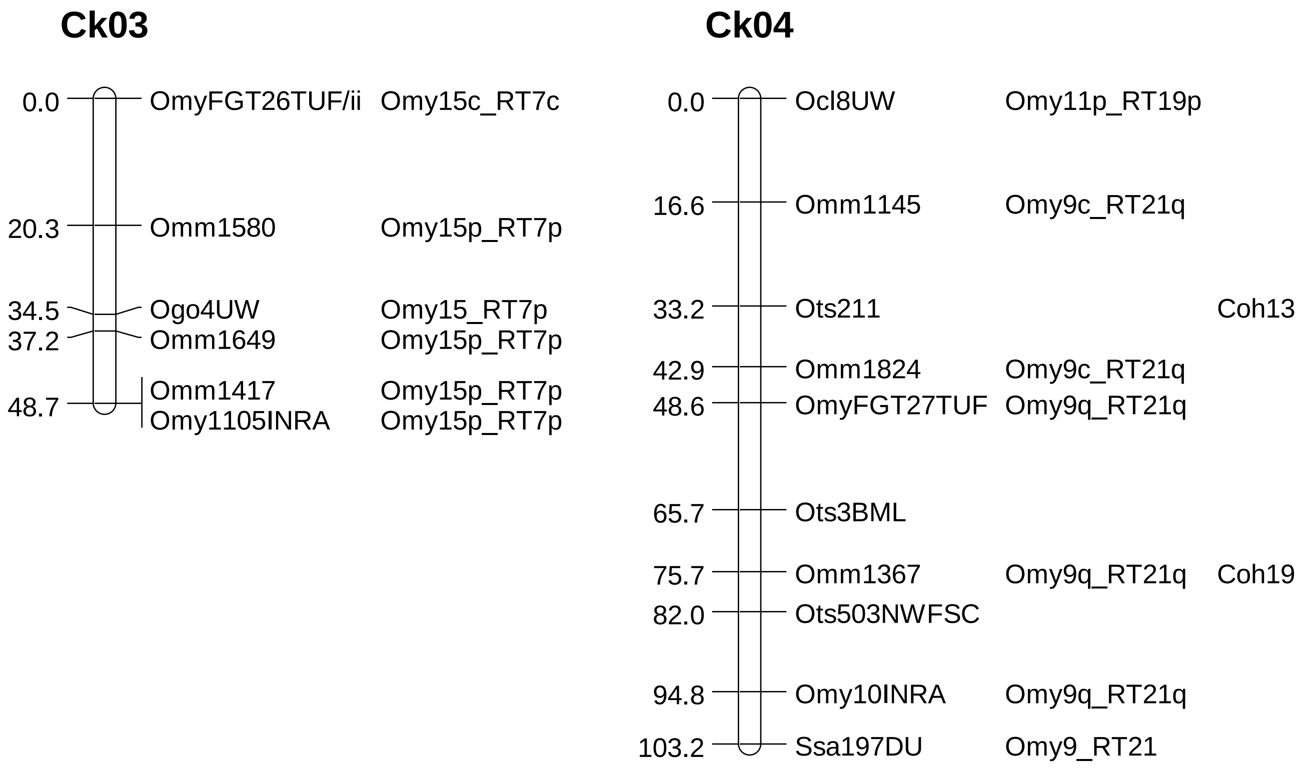
Ck03
0.0 OmyFGT26TUF/ii Omy15c_RT7c
20.3 Omm1580 Omy15p_RT7p
34.5 Ogo4UW Omy15_RT7p
37.2 Omm1649 Omy15p_RT7p
48.7 Omm1417 Omy15p_RT7p
Omy1105INRA Omy15p_RT7p
Ck04
0.0 Ocl8UW Omy11p_RT19p
16.6 Omm1145 Omy9c_RT21q
33.2 Ots211 Coh13
42.9 Omm1824 Omy9c_RT21q
48.6 OmyFGT27TUF Omy9q_RT21q
65.7 Ots3BML
75.7 Omm1367 Omy9q_RT21q Coh19
82.0 Ots503NWFSC
94.8 Omy10INRA Omy9q_RT21q
103.2 Ssa197DU Omy9_RT21

Ck05

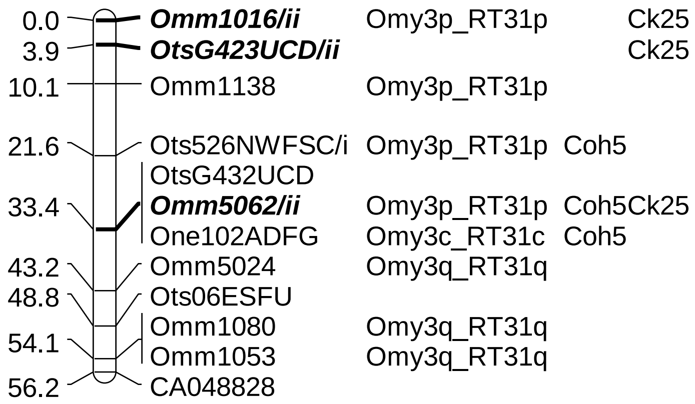
0.0 Omm1016/ii Omy3p_RT31p Ck25
3.9 OtsG423UCD/ii Ck25
10.1 Omm1138 Omy3p_RT31p
21.6 Ots526NWFSC/i Omy3p_RT31p Coh5
OtsG432UCD
33.4 Omm5062/ii Omy3p_RT31p Coh5Ck25
One102ADFG Omy3c_RT31c Coh5
43.2 Omm5024 Omy3q_RT31q
48.8 Ots06ESFU
54.1 Omm1080 Omy3q_RT31q
Omm1053 Omy3q_RT31q
56.2 CA048828

Ck06

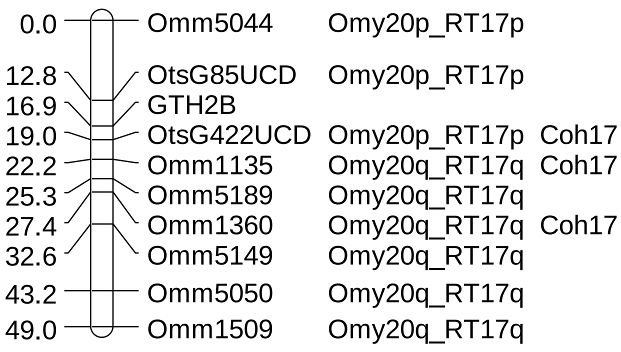
0.0 Omm5044 Omy20p_RT17p
12.8 OtsG85UCD Omy20p_RT17p
16.9 GTH2B
19.0 OtsG422UCD Omy20p_RT17p Coh17
22.2 Omm1135 Omy20q_RT17q Coh17
25.3 Omm5189 Omy20q_RT17q
27.4 Omm1360 Omy20q_RT17q Coh17
32.6 Omm5149 Omy20q_RT17q
43.2 Omm5050 Omy20q_RT17q
49.0 Omm1509 Omy20q_RT17q

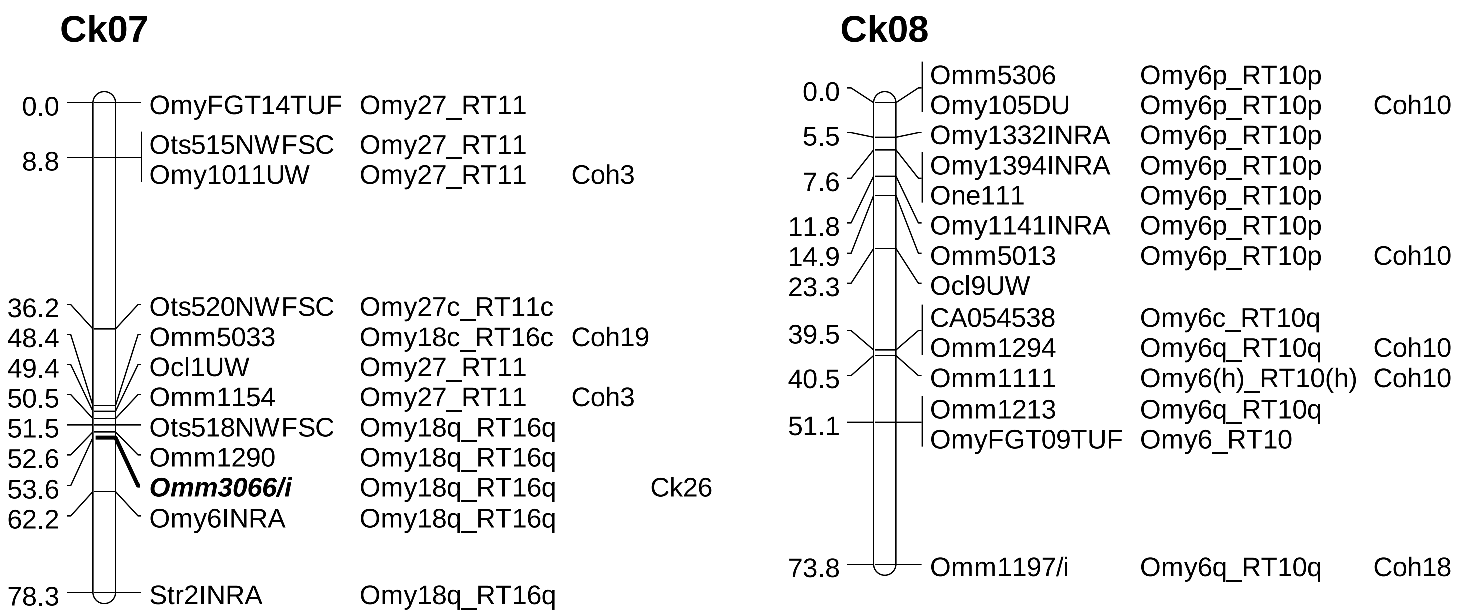
Ck07
0.0 OmyFGT14TUF Omy27_RT11
8.8 Ots515NWFSC Omy27_RT11
Omy1011UW Omy27_RT11 Coh3
36.2 Ots520NWFSC Omy27c_RT11c
48.4 Omm5033 Omy18c_RT16c Coh19
49.4 Ocl1UW Omy27_RT11
50.5 Omm1154 Omy27_RT11 Coh3
51.5 Ots518NWFSC Omy18q_RT16q
52.6 Omm1290 Omy18q_RT16q
53.6 Omm3066/i Omy18q_RT16q Ck26
62.2 Omy6INRA Omy18q_RT16q
78.3 Str2INRA Omy18q_RT16q
Ck08
0.0 Omm5306 Omy6p_RT10p
Omy105DU Omy6p_RT10p Coh10
5.5 Omy1332INRA Omy6p_RT10p
7.6 Omy1394INRA Omy6p_RT10p
One111 Omy6p_RT10p
11.8 Omy1141INRA Omy6p_RT10p
14.9 Omm5013 Omy6p_RT10p Coh10
23.3 Ocl9UW
39.5 CA054538 Omy6c_RT10q
Omm1294 Omy6q_RT10q Coh10
40.5 Omm1111 Omy6(h)_RT10(h) Coh10
51.1 Omm1213 Omy6q_RT10q
OmyFGT09TUF Omy6_RT10
73.8 Omm1197/i Omy6q_RT10q Coh18

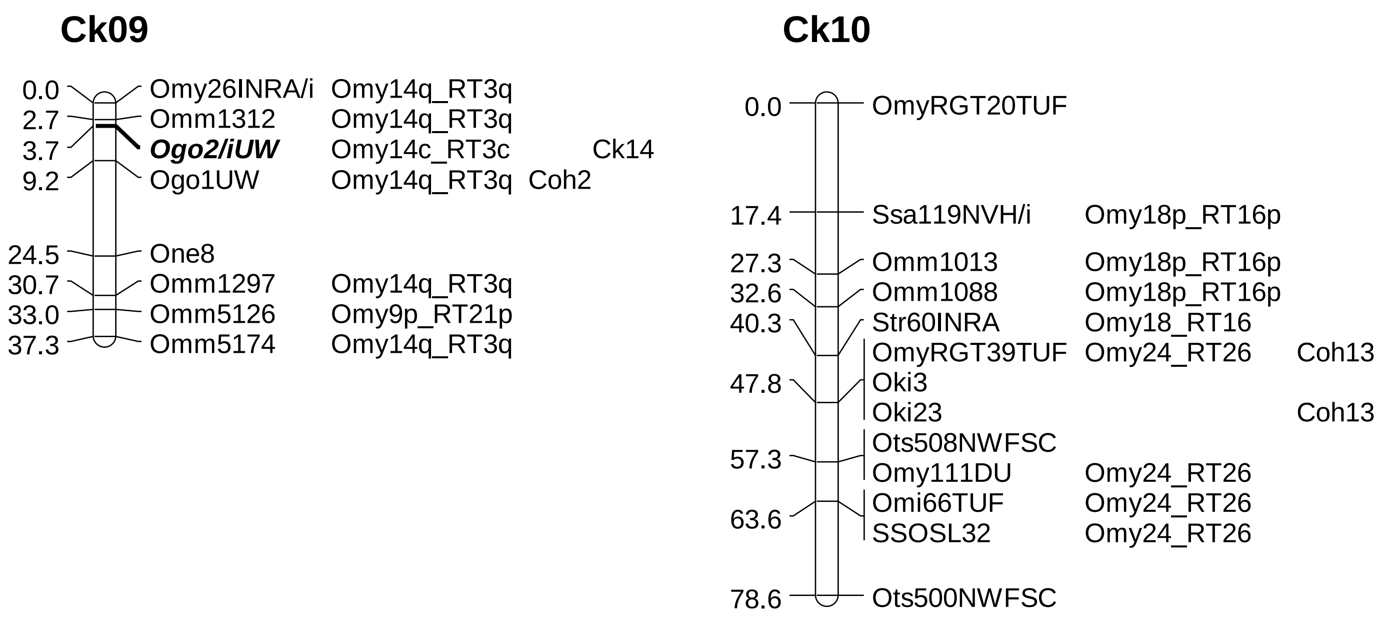
Ck09
0.0 Omy26INRA/i Omy14q_RT3q
2.7 Omm1312 Omy14q_RT3q
3.7 Ogo2/iUW Omy14c_RT3c Ck14
9.2 Ogo1UW Omy14q_RT3q Coh2
24.5 One8
30.7 Omm1297 Omy14q_RT3q
33.0 Omm5126 Omy9p_RT21p
37.3 Omm5174 Omy14q_RT3q
Ck10
0.0 OmyRGT20TUF
17.4 Ssa119NVH/i Omy18p_RT16p
27.3 Omm1013 Omy18p_RT16p
32.6 Omm1088 Omy18p_RT16p
40.3 Str60INRA Omy18_RT16
OmyRGT39TUF Omy24_RT26 Coh13
47.8 Oki3
Oki23 Coh13
Ots508NWFSC
57.3 Omy111DU Omy24_RT26
Omi66TUF Omy24_RT26
63.6 SSOSL32 Omy24_RT26
78.6 Ots500NWFSC

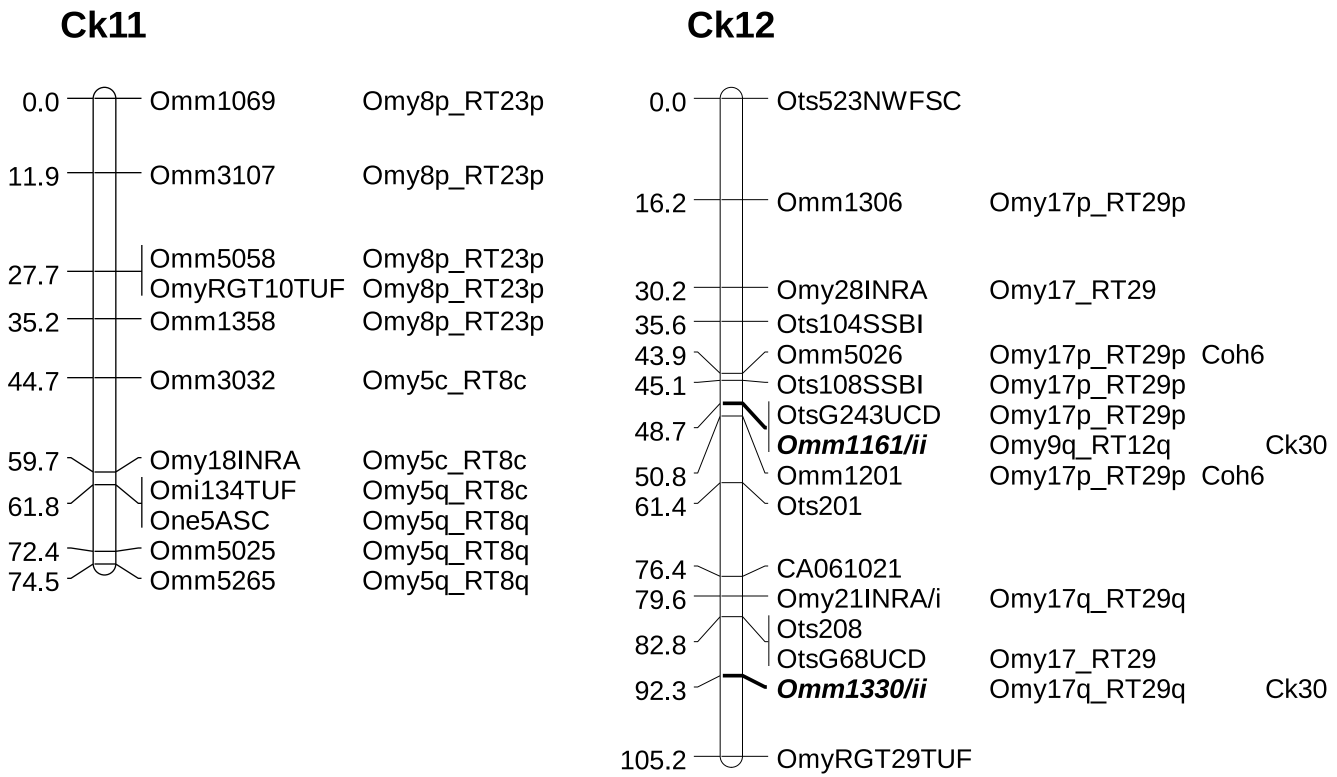
Ck11
0.0 Omm1069 Omy8p_RT23p
11.9 Omm3107 Omy8p_RT23p
27.7 Omm5058 Omy8p_RT23p
OmyRGT10TUF Omy8p_RT23p
35.2 Omm1358 Omy8p_RT23p
44.7 Omm3032 Omy5c_RT8c
59.7 Omy18INRA Omy5c_RT8c
61.8 Omi134TUF Omy5q_RT8c
One5ASC Omy5q_RT8q
72.4 Omm5025 Omy5q_RT8q
74.5 Omm5265 Omy5q_RT8q
Ck12
0.0 Ots523NWFSC
16.2 Omm1306 Omy17p_RT29p
30.2 Omy28INRA Omy17_RT29
35.6 Ots104SSBI
43.9 Omm5026 Omy17p_RT29p Coh6
45.1 Ots108SSBI Omy17p_RT29p
48.7 OtsG243UCD Omy17p_RT29p
Omm1161/ii Omy9q_RT12q Ck30
50.8 Omm1201 Omy17p_RT29p Coh6
61.4 Ots201
76.4 CA061021
79.6 Omy21INRA/i Omy17q_RT29q
82.8 Ots208
OtsG68UCD Omy17_RT29
92.3 Omm1330/ii Omy17q_RT29q Ck30
105.2 OmyRGT29TUF

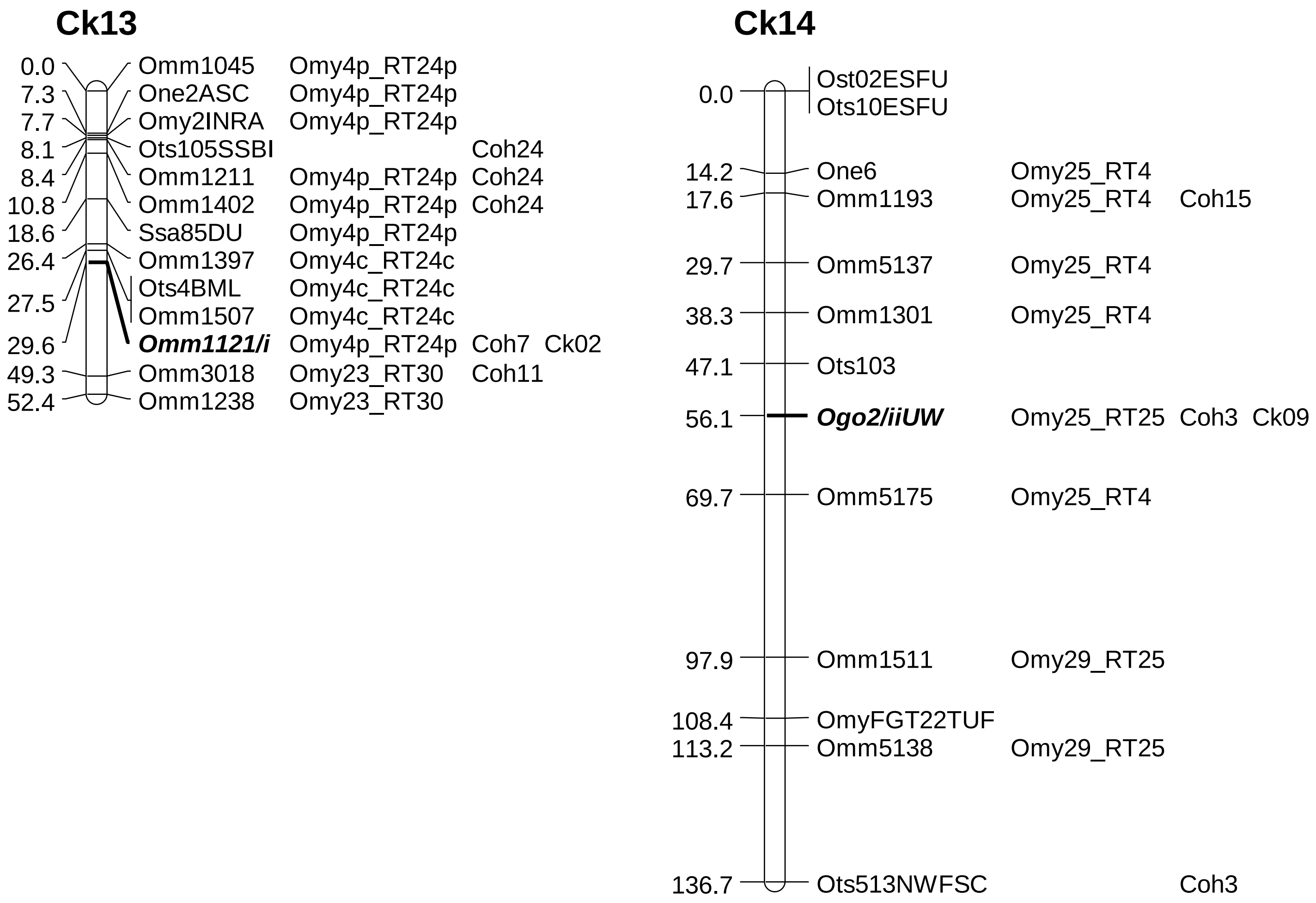
Ck13
0.0 Omm1045 Omy4p_RT24p
7.3 One2ASC Omy4p_RT24p
7.7 Omy2INRA Omy4p_RT24p
8.1 Ots105SSBI Coh24
8.4 Omm1211 Omy4p_RT24p Coh24
10.8 Omm1402 Omy4p_RT24p Coh24
18.6 Ssa85DU Omy4p_RT24p
26.4 Omm1397 Omy4c_RT24c
27.5 Ots4BML Omy4c_RT24c
Omm1507 Omy4c_RT24c
29.6 Omm1121/i Omy4p_RT24p Coh7 Ck02
49.3 Omm3018 Omy23_RT30 Coh11
52.4 Omm1238 Omy23_RT30
Ck14
0.0 Ost02ESFU
Ots10ESFU
14.2 One6 Omy25_RT4
17.6 Omm1193 Omy25_RT4 Coh15
29.7 Omm5137 Omy25_RT4
38.3 Omm1301 Omy25_RT4
47.1 Ots103
56.1 Ogo2/iiUW Omy25_RT25 Coh3 Ck09
69.7 Omm5175 Omy25_RT4
97.9 Omm1511 Omy29_RT25
108.4 OmyFGT22TUF
113.2 Omm5138 Omy29_RT25
136.7 Ots513NWFSC Coh3

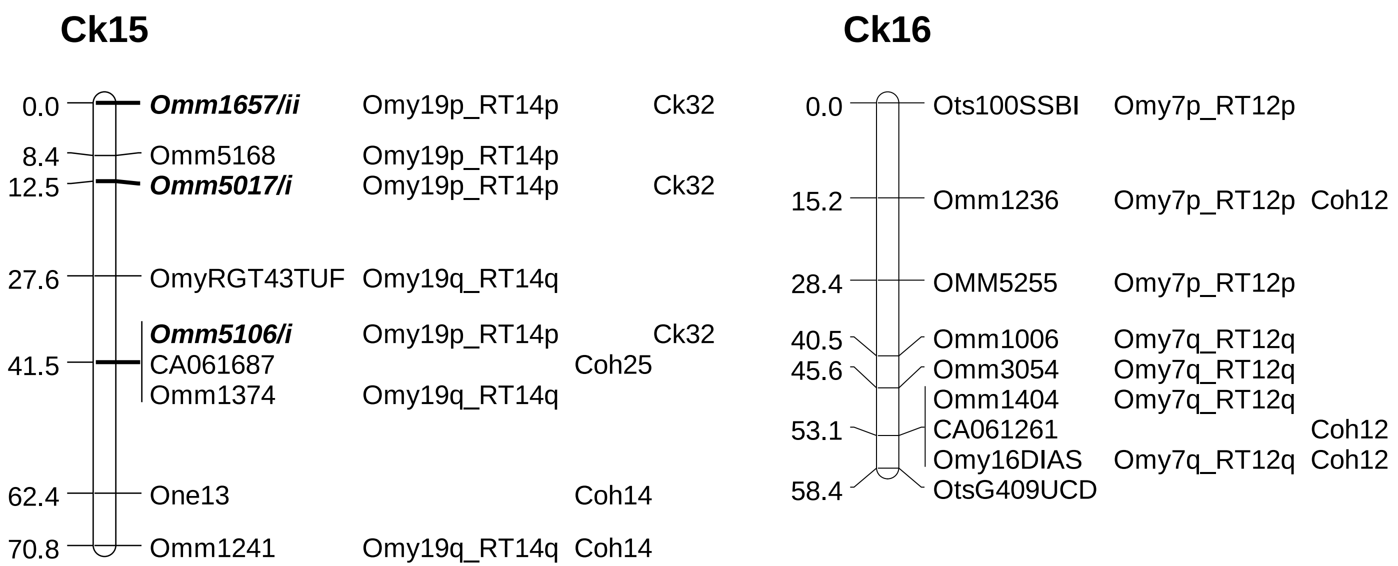
Ck15
0.0 Omm1657/ii Omy19p_RT14p Ck32
8.4 Omm5168 Omy19p_RT14p
12.5 Omm5017/i Omy19p_RT14p Ck32
27.6 OmyRGT43TUF Omy19q_RT14q
Omm5106/i Omy19p_RT14p Ck32
41.5 CA061687 Coh25
Omm1374 Omy19q_RT14q
62.4 One13 Coh14
70.8 Omm1241 Omy19q_RT14q Coh14
Ck16
0.0 Ots100SSBI Omy7p_RT12p
15.2 Omm1236 Omy7p_RT12p Coh12
28.4 OMM5255 Omy7p_RT12p
40.5 Omm1006 Omy7q_RT12q
45.6 Omm3054 Omy7q_RT12q
Omm1404 Omy7q_RT12q
53.1 CA061261 Coh12
Omy16DIAS Omy7q_RT12q Coh12
58.4 OtsG409UCD

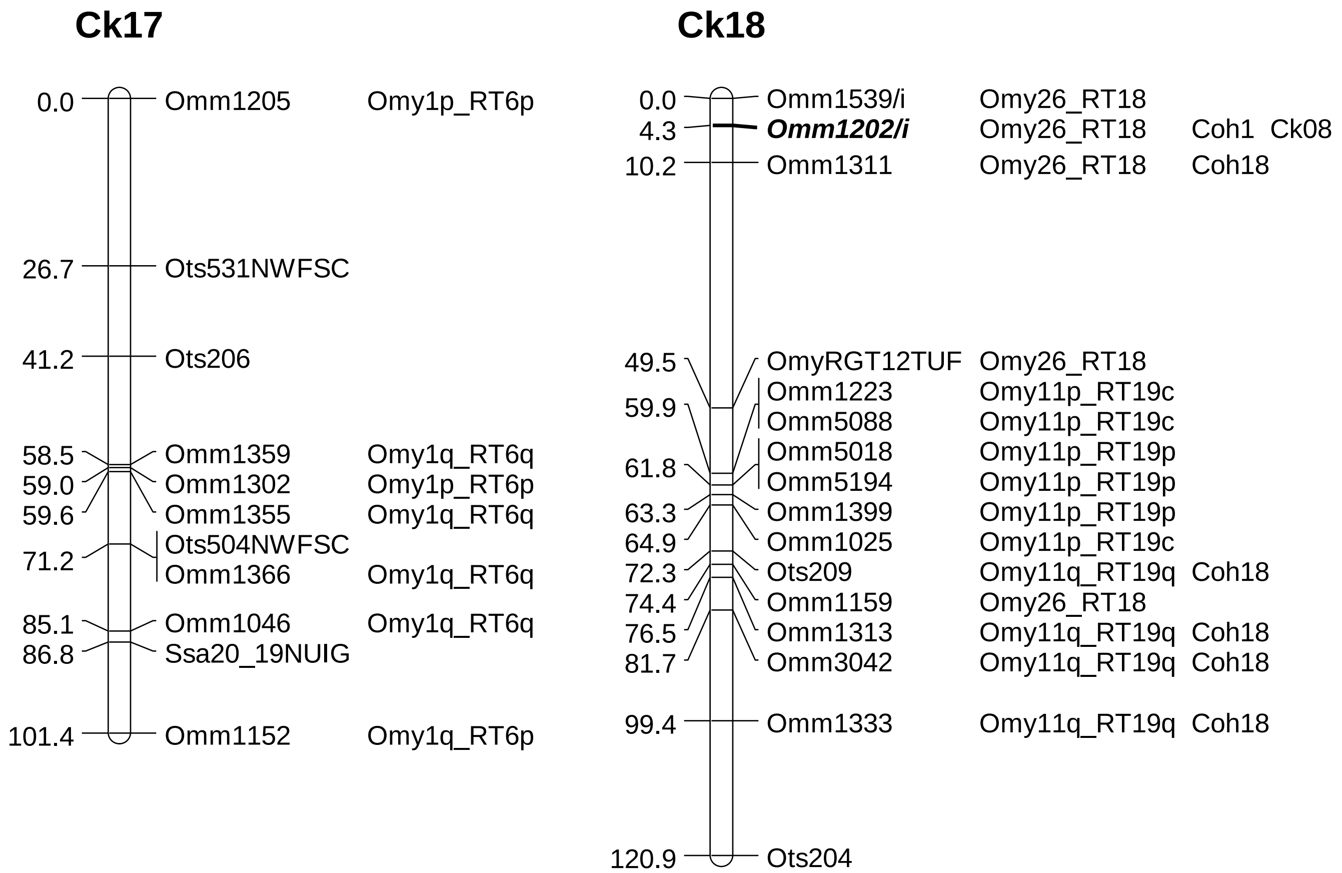
Ck17
0.0 Omm1205 Omy1p_RT6p
26.7 Ots531NWFSC
41.2 Ots206
58.5 Omm1359 Omy1q_RT6q
59.0 Omm1302 Omy1p_RT6p
59.6 Omm1355 Omy1q_RT6q
71.2 Ots504NWFSC
Omm1366 Omy1q_RT6q
85.1 Omm1046 Omy1q_RT6q
86.8 Ssa20_19NUIG
101.4 Omm1152 Omy1q_RT6p
Ck18
0.0 Omm1539/i Omy26_RT18
4.3 Omm1202/i Omy26_RT18 Coh1 Ck08
10.2 Omm1311 Omy26_RT18 Coh18
49.5 OmyRGT12TUF Omy26_RT18
Omm1223 Omy11p_RT19c
59.9 Omm5088 Omy11p_RT19c
Omm5018 Omy11p_RT19p
61.8 Omm5194 Omy11p_RT19p
63.3 Omm1399 Omy11p_RT19p
64.9 Omm1025 Omy11p_RT19c
72.3 Ots209 Omy11q_RT19q Coh18
74.4 Omm1159 Omy26_RT18
76.5 Omm1313 Omy11q_RT19q Coh18
81.7 Omm3042 Omy11q_RT19q Coh18
99.4 Omm1333 Omy11q_RT19q Coh18
120.9 Ots204

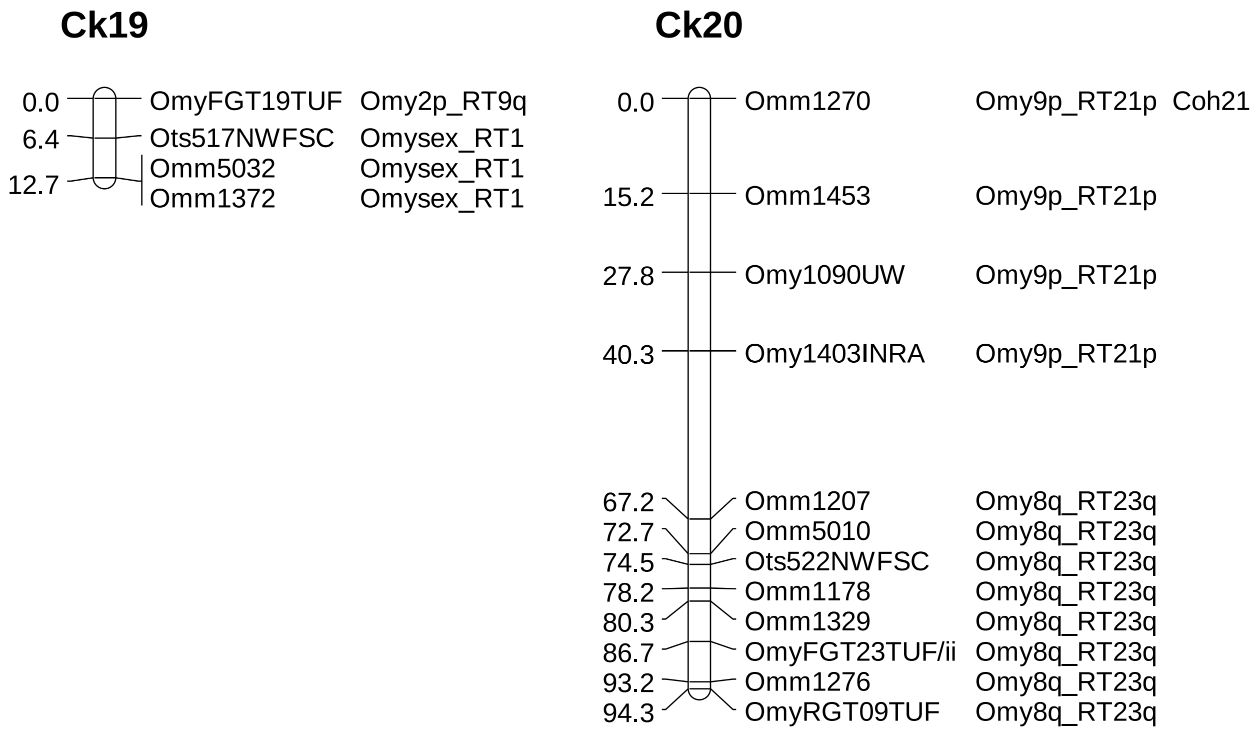
Ck19
0.0 OmyFGT19TUF Omy2p_RT9q
6.4 Ots517NWFSC Omysex_RT1
12.7 Omm5032 Omysex_RT1
Omm1372 Omysex_RT1
Ck20
0.0 Omm1270 Omy9p_RT21p Coh21
15.2 Omm1453 Omy9p_RT21p
27.8 Omy1090UW Omy9p_RT21p
40.3 Omy1403INRA Omy9p_RT21p
67.2 Omm1207 Omy8q_RT23q
72.7 Omm5010 Omy8q_RT23q
74.5 Ots522NWFSC Omy8q_RT23q
78.2 Omm1178 Omy8q_RT23q
80.3 Omm1329 Omy8q_RT23q
86.7 OmyFGT23TUF/ii Omy8q_RT23q
93.2 Omm1276 Omy8q_RT23q
94.3 OmyRGT09TUF Omy8q_RT23q

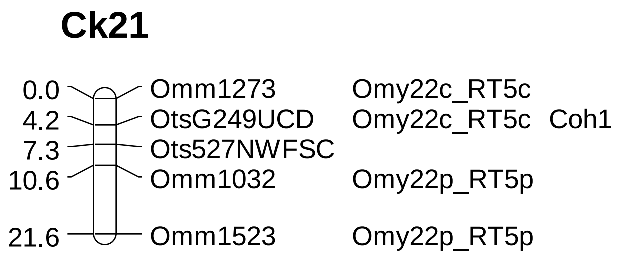
Ck21
0.0
4.2
7.3
10.6
21.6
Omm1273 Omy22c_RT5c
OtsG249UCD Omy22c_RT5c Coh1
Ots527NWFSC
Omm1032 Omy22p_RT5p
Omm1523 Omy22p_RT5p

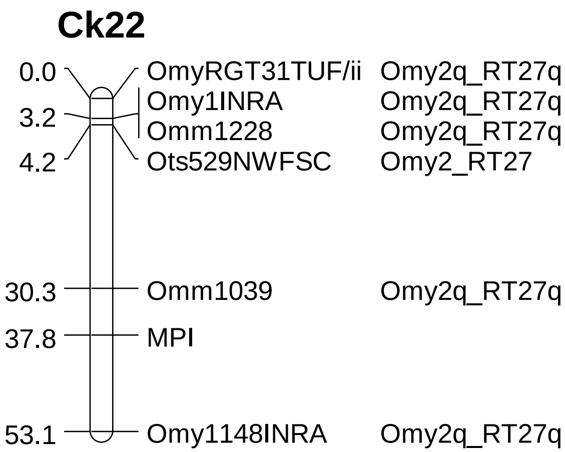
Ck22
0.0
3.2
4.2
30.3
37.8
53.1
OmyRGT31TUF/ii Omy2q_RT27q
Omy1INRA Omy2q_RT27q
Omm1228 Omy2q_RT27q
Ots529NWFSC Omy2_RT27
Omm1039 Omy2q_RT27q
MPI
Omy1148INRA Omy2q_RT27q

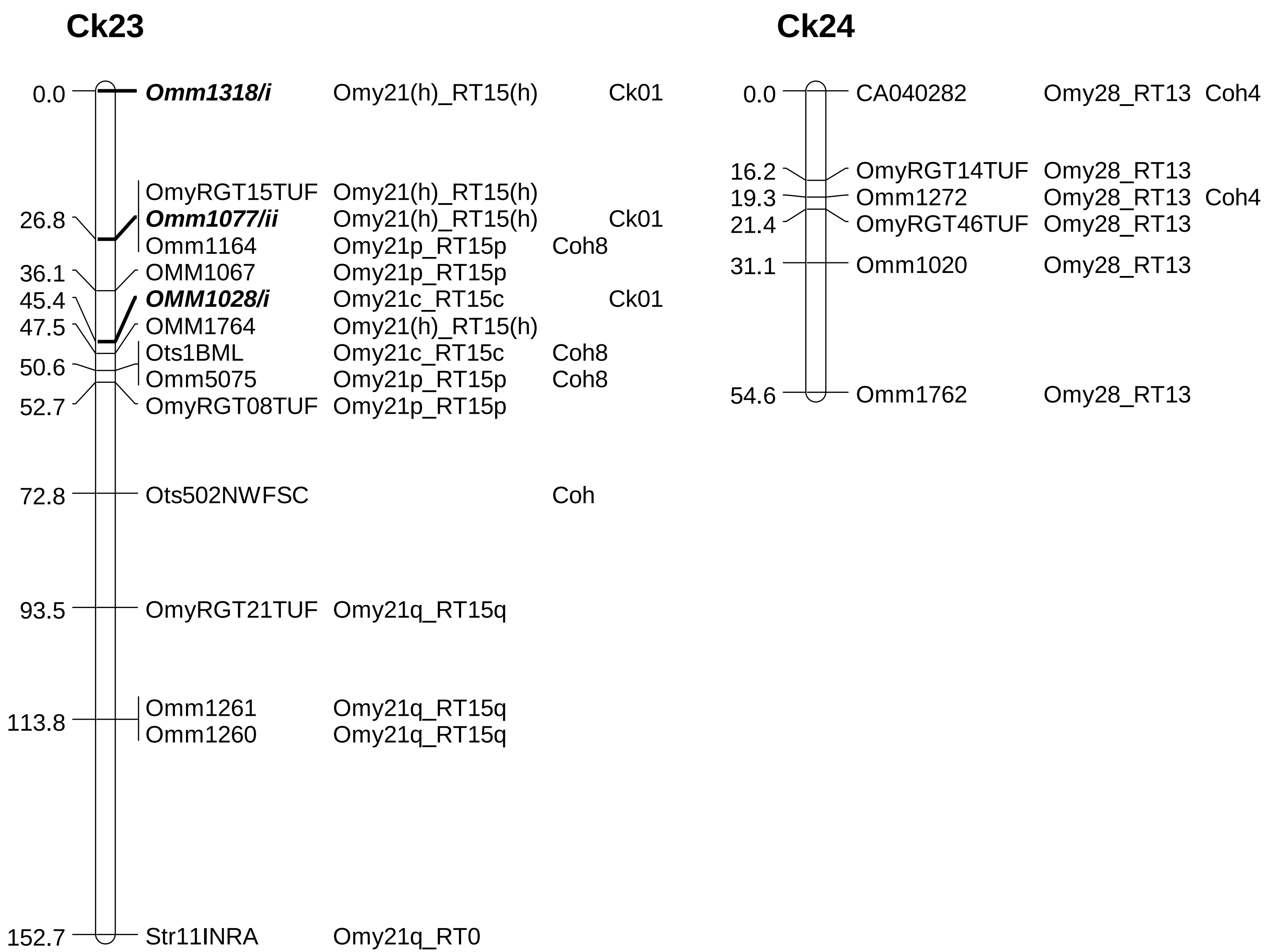
Ck23
0.0 Omm1318/i Omy21(h)_RT15(h) Ck01
OmyRGT15TUF Omy21(h)_RT15(h)
26.8 Omm1077/ii Omy21(h)_RT15(h) Ck01
Omm1164 Omy21p_RT15p Coh8
36.1 OMM1067 Omy21p_RT15p
45.4 OMM1028/i Omy21c_RT15c Ck01
47.5 OMM1764 Omy21(h)_RT15(h)
Ots1BML Omy21c_RT15c Coh8
50.6 Omm5075 Omy21p_RT15p Coh8
52.7 OmyRGT08TUF Omy21p_RT15p
72.8 Ots502NWFSC Coh
93.5 OmyRGT21TUF Omy21q_RT15q
113.8 Omm1261 Omy21q_RT15q
Omm1260 Omy21q_RT15q
152.7 Str11INRA Omy21q_RT0
Ck24
0.0 CA040282 Omy28_RT13 Coh4
16.2 OmyRGT14TUF Omy28_RT13
19.3 Omm1272 Omy28_RT13 Coh4
21.4 OmyRGT46TUF Omy28_RT13
31.1 Omm1020 Omy28_RT13
54.6 Omm1762 Omy28_RT13

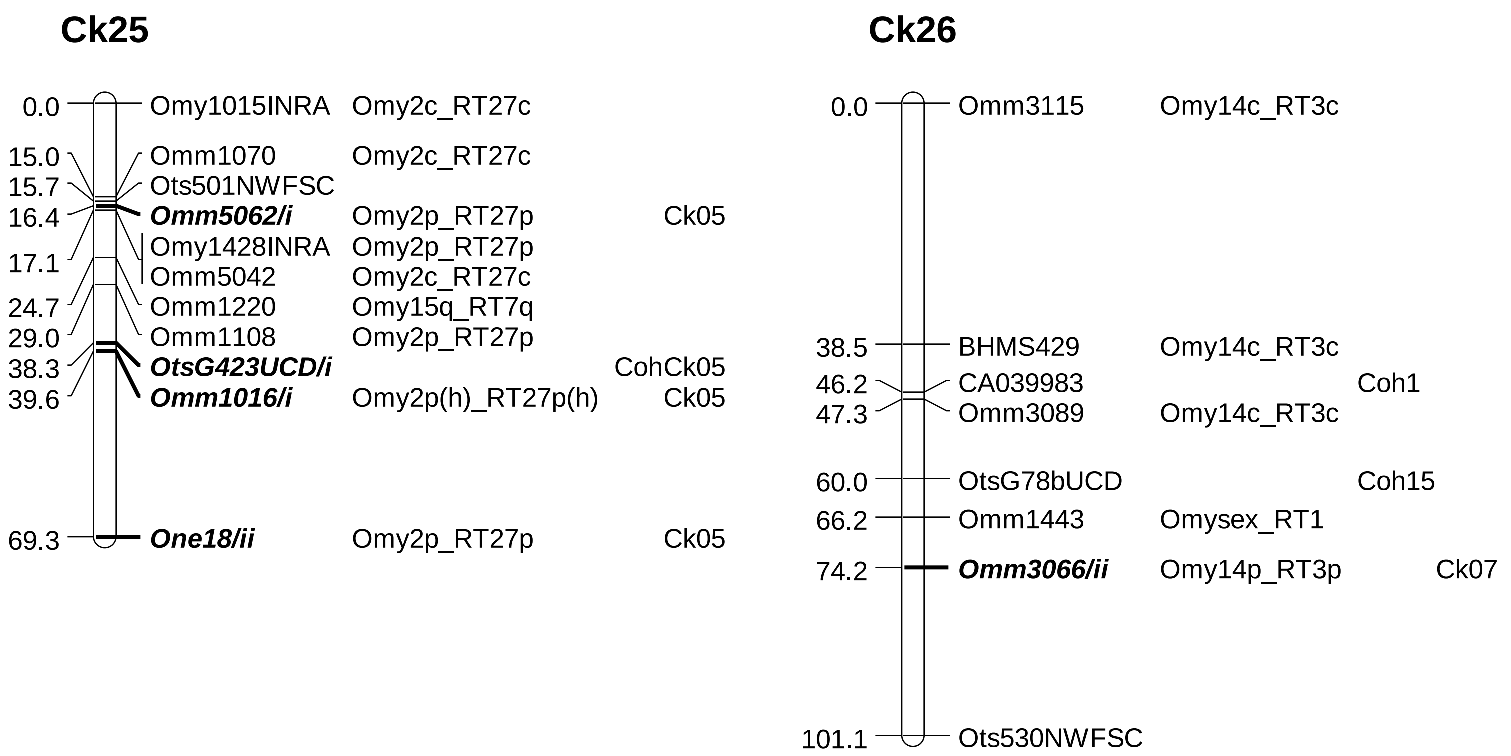
Ck25
0.0 Omy1015INRA Omy2c_RT27c
15.0 Omm1070 Omy2c_RT27c
15.7 Ots501NWFSC
16.4 Omm5062/i Omy2p_RT27p Ck05
17.1 Omy1428INRA Omy2p_RT27p
Omm5042 Omy2c_RT27c
24.7 Omm1220 Omy15q_RT7q
29.0 Omm1108 Omy2p_RT27p
38.3 OtsG423UCD/i CohCk05
39.6 Omm1016/i Omy2p(h)_RT27p(h) Ck05
69.3 One18/ii Omy2p_RT27p Ck05
Ck26
0.0 Omm3115 Omy14c_RT3c
38.5 BHMS429 Omy14c_RT3c
46.2 CA039983 Coh1
47.3 Omm3089 Omy14c_RT3c
60.0 OtsG78bUCD Coh15
66.2 Omm1443 Omysex_RT1
74.2 Omm3066/ii Omy14p_RT3p Ck07
101.1 Ots530NWFSC

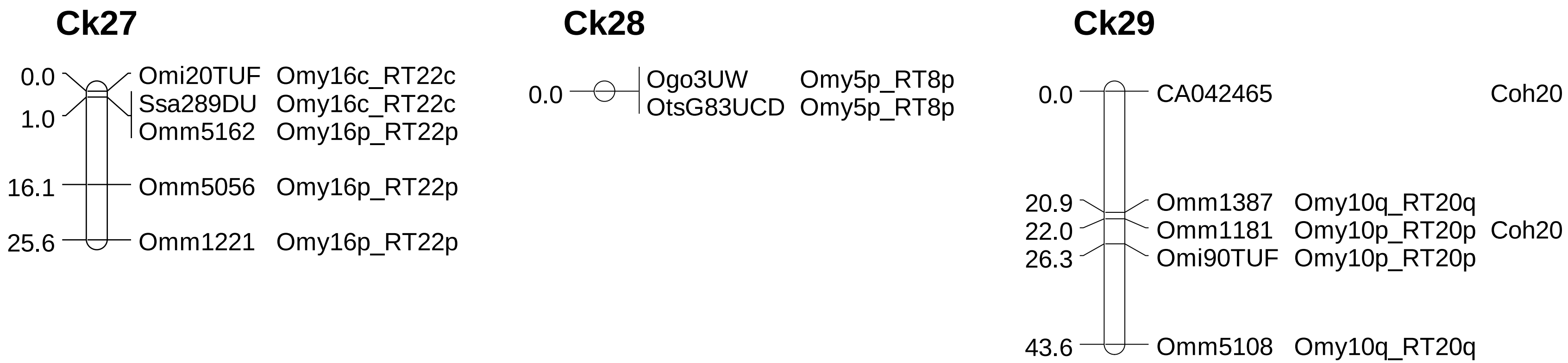
Ck27
0.0 Omi20TUF Omy16c_RT22c
Ssa289DU Omy16c_RT22c
1.0 Omm5162 Omy16p_RT22p
16.1 Omm5056 Omy16p_RT22p
25.6 Omm1221 Omy16p_RT22p
Ck28
0.0 Ogo3UW Omy5p_RT8p
OtsG83UCD Omy5p_RT8p
Ck29
0.0 CA042465 Coh20
20.9 Omm1387 Omy10q_RT20q
22.0 Omm1181 Omy10p_RT20p Coh20
26.3 Omi90TUF Omy10p_RT20p
43.6 Omm5108 Omy10q_RT20q

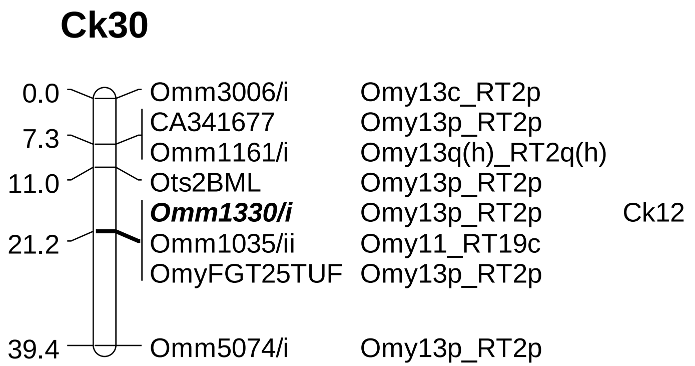
Ck30
0.0
7.3
11.0
21.2
39.4
Omm3006/i Omy13c_RT2p
CA341677 Omy13p_RT2p
Omm1161/i Omy13q(h)_RT2q(h)
Ots2BML Omy13p_RT2p
Omm1330/i Omy13p_RT2p Ck12
Omm1035/ii Omy11_RT19c
OmyFGT25TUF Omy13p_RT2p
Omm5074/i Omy13p_RT2p

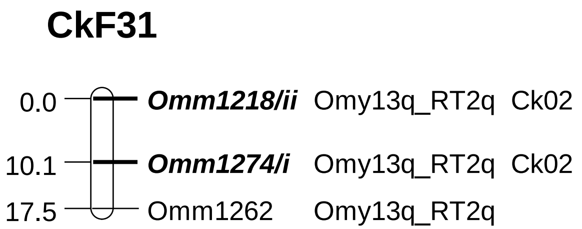
CkF31
0.0
10.1
17.5
Omm1218/ii Omy13q_RT2q Ck02
Omm1274/i Omy13q_RT2q Ck02
Omm1262 Omy13q_RT2q

CkM31

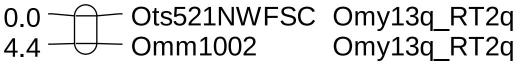
0.0 Ots521NWFSC Omy13q_RT2q
4.4 Omm1002 Omy13q_RT2q

Ck32

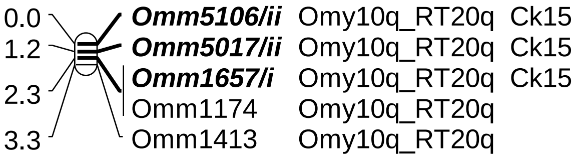
0.0 *Omm5106/ii* Omy10q_RT20q Ck15
1.2 *Omm5017/ii* Omy10q_RT20q Ck15
2.3 *Omm1657/i* Omy10q_RT20q Ck15
3.3 Omm1174 Omy10q_RT20q
Omm1413 Omy10q_RT20q

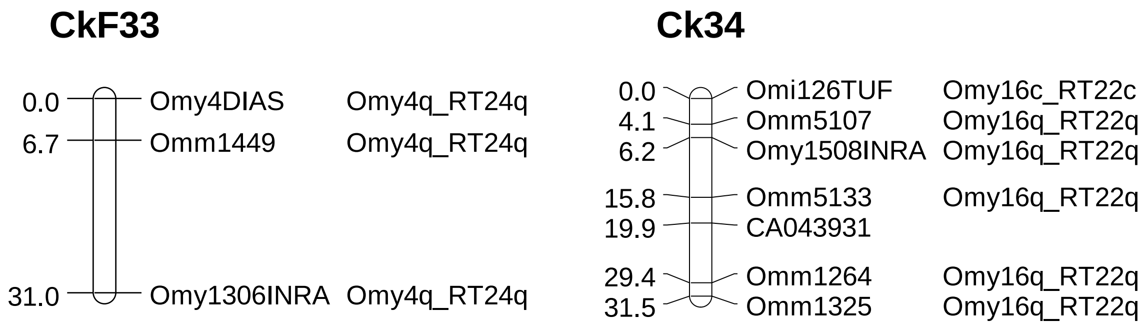

Figure S1 Graphic representation of linkage groups mapped in Chinook salmon. The map is a consensus of marker order and distances between the female and male parents. Recombination distances are in centiMorgan (Kosambi). Loci are annotated with the chromosome (Omy) or linkage group (RT) assignment in rainbow trout. Rainbow trout chromosome arms (p or q) or centromere positions (c) are identified where possible. Markers that mapped to the homoelog in rainbow trout are designated (h). Duplicated loci (bold) are identified as/i or /ii and annotated with the homeologous Chinook linkage groups where both loci are mapped.
